# Optimizing Magnesium Uptake in *Lacticaseibacillus Rhamnosus* To Advance Nutribiotic Strategies

**DOI:** 10.1007/s00284-026-04721-8

**Published:** 2026-01-12

**Authors:** Rodica-Anita Varvara, Heike Budde, Ruth Ley, Dan Cristian Vodnar

**Affiliations:** 1 Department of Food Science and Technology, Life Science Institute, University of Agricultural Sciences and Veterinary Medicine, Calea Mănăștur 3-5, Cluj-Napoca, 400372 Romania; 2 Department of Microbiome Science, Max Planck Institute for Biology, 72076 Tübingen, Germany

## Abstract

Magnesium is an essential mineral involved in more than 300 enzymatic reactions, including protein synthesis, neuromuscular function, and blood pressure regulation. Recent studies have highlighted the role of probiotics, beneficial microorganisms in the human gut, in enhancing mineral absorption. However, the interaction between magnesium and probiotic strains remains poorly understood. This study aimed to investigate the capacity of *Lacticaseibacillus rhamnosus* ATCC 53,103 to uptake and internalize magnesium ions, using MgSO₄ as a supplementation source. The bacterium was cultivated in MRS medium with varying concentrations of MgSO₄ (0, 0.1444, 0.722, and 1.444 g/L) over seven days. Quantitative analysis revealed that at 0.722 g/L MgSO₄, intracellular magnesium accumulation peaked at 0.7 mg/dL by Day 3, representing a 7-fold increase compared to the control (0.1 mg/dL). Scanning and transmission electron microscopy indicated essential morphological changes, including ruffled cell surfaces and enhanced ribosomal visibility. These findings suggest that *L. rhamnosus* can internalize magnesium under enriched conditions, supporting its potential as a microbial carrier for nutribiotic applications. This work contributes to the growing field of mineral–microbe interactions and may inform the development of probiotic-based strategies to improve magnesium bioaccessibility in the human gut.

**Graphical Abstract:**

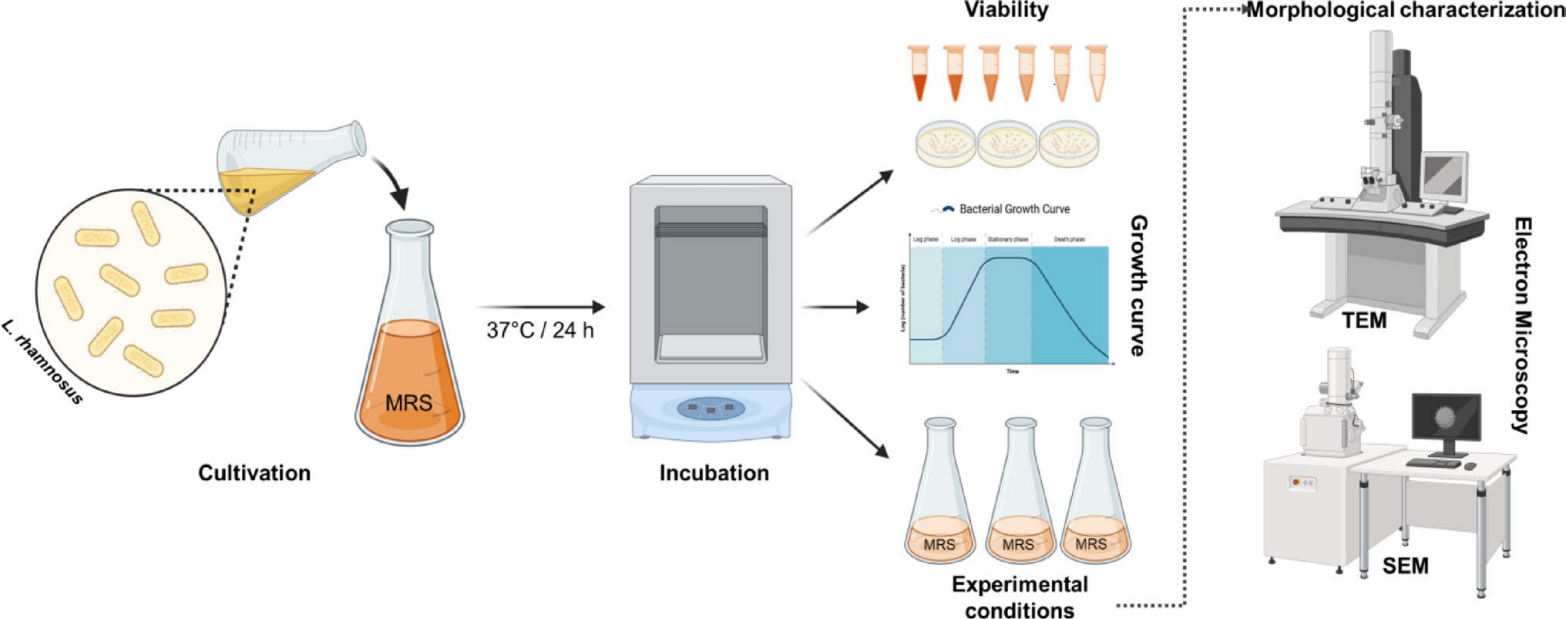

**Supplementary Information:**

The online version contains supplementary material available at 10.1007/s00284-026-04721-8.

## Introduction

 Magnesium (Mg²⁺) is an essential mineral involved in more than 300 enzymatic reactions that support muscle and nerve function, blood pressure regulation, bone strength, and mental well-being [1–4]. It plays a key role in energy metabolism, influencing insulin and glucose homeostasis [5]. The human body contains about 25–35 g of Mg²⁺, with 53% stored in bones, 46% in muscles and soft tissues, and only 1% in plasma [2]. Despite its importance, up to 75% of the U.S. population fails to meet the recommended daily intake (400–420 mg for men and 300–320 mg for women) [6], mainly due to diets high in processed foods, certain medical conditions, and the use of medications or substances such as alcohol, sugar, caffeine, and diuretics [7]. Mg²⁺ deficiency may cause fatigue, irritability, muscle cramps, and insomnia, progressing in severe cases to arrhythmia, anxiety, and increased risk of cardiovascular disease, diabetes, osteoporosis, and chronic inflammation that can further disrupt gut microbiota [3, 8, 9].

Ensuring adequate Mg²⁺ intake from bioavailable and safe sources—such as green leafy vegetables, nuts, seeds, and whole grains—is vital for maintaining optimal health. However, conventional supplementation often suffers from limited intestinal absorption and poor retention. In recent years, the concept of **nutribiotics** has gained attention as an innovative nutritional strategy. The term refers to beneficial microorganisms or their derivatives with *nutritional* functions rather than therapeutic roles, including probiotics and related biotics that enhance nutrient bioaccessibility and absorption [10, 11]. In this context, probiotics can serve as biological carriers of minerals such as Mg²⁺, thereby improving their bioavailability and functionality [12]. Metal ions such as Mg²⁺ are more efficiently tolerated and absorbed when associated with microbial cell components (e.g., as metalloproteins). Within lactic acid bacteria (LAB), Mg²⁺ supports enzyme production and cellular function. Probiotic *Lacticaseibacillus* strains, classified as generally recognized as safe (GRAS), are extensively used in fermented foods [13]. Their cell walls, rich in teichoic acids and peptidoglycan, facilitate the biosorption of metal ions through mechanisms such as ion exchange, chelation, and microprecipitation [12, 13]. Once binding sites are saturated, Mg²⁺ can accumulate intracellularly until equilibrium is reached.

Although the interaction between Mg²⁺ and probiotics remains underexplored, evidence suggests that probiotics can enhance mineral absorption and influence host metabolism. For instance, supplementation with *Lacticaseibacillus rhamnosus* HN001 improved calcium and magnesium uptake and bone density in rats [14]. This effect is likely mediated by probiotic-derived short-chain fatty acids (SCFAs), which lower intestinal pH, increase mineral solubility, and stimulate epithelial proliferation [15, 16]. Probiotics may also modulate intestinal motility and tight junction integrity, thereby enhancing nutrient transport [16–18]. Interestingly, Mg²⁺ deficiency has been linked to reduced microbial diversity and anxiety-like behavior, highlighting a bidirectional Mg²⁺–microbiota relationship [19]. Despite growing evidence of mineral–microbe interactions, few studies have examined the potential of probiotics as *nutribiotic carriers* for magnesium delivery. The synergistic relationship between probiotics and Mg²⁺ represents a promising approach to improve mineral bioavailability and address widespread Mg²⁺ deficiency [20, 21].

**Fig. 1:**
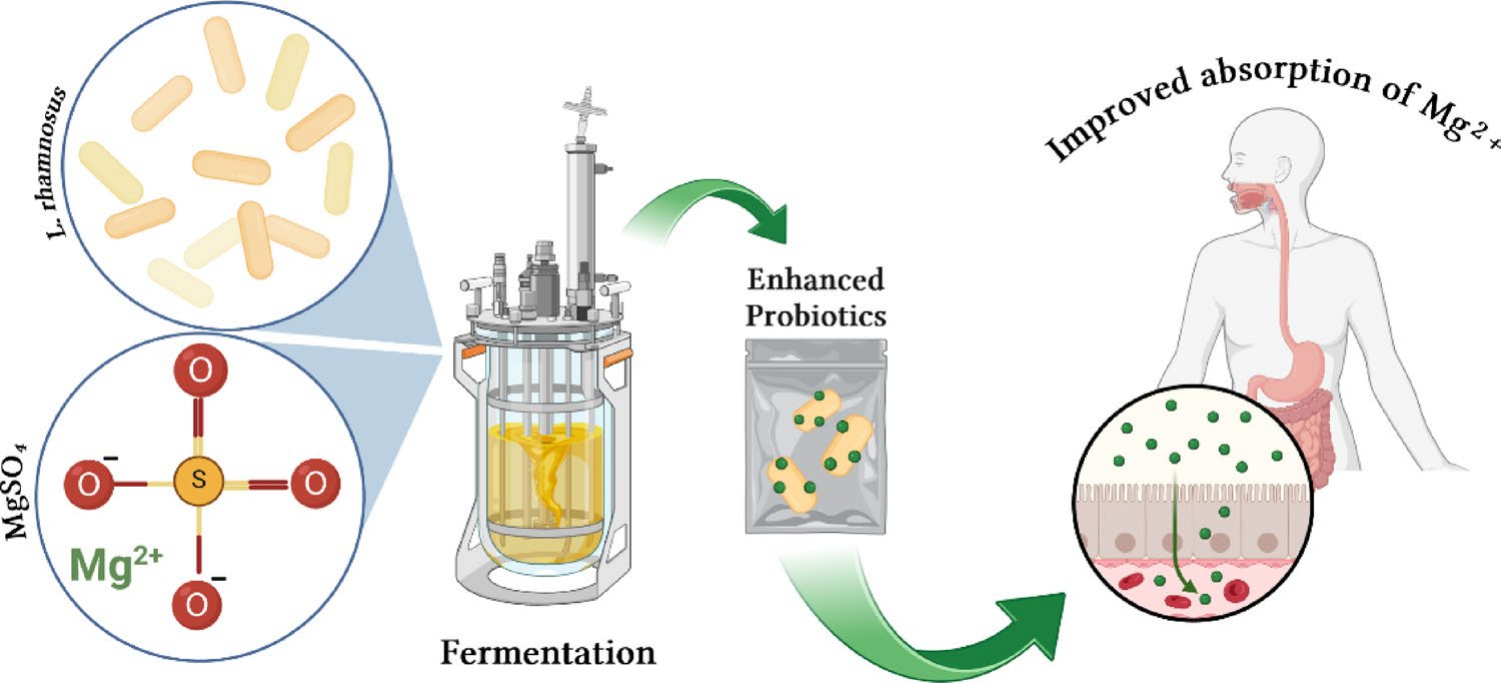
Conceptual pathway of *Lacticaseibacillus rhamnosus* fermentation with MgSO₄ for improved magnesium bioaccessibility and nutribiotic development. Created using BioRender.com

The present study investigates the ability of *Lacticaseibacillus rhamnosus* to internalize Mg²⁺ during fermentation with MgSO₄ supplementation, to enhance its nutribiotic potential. We further explore how such Mg-enriched probiotics may improve magnesium bioaccessibility and absorption in the gastrointestinal tract. This work lays the foundation for optimizing microbial uptake pathways and developing next-generation functional probiotics capable of delivering bioavailable micronutrients [21]. Figure 1 illustrates the conceptual framework of this study: *L. rhamnosus* is cultivated in a Mg²⁺-supplemented medium to produce magnesium-enriched probiotic cells (“enhanced probiotics”), which can subsequently enhance magnesium bioaccessibility and absorption in the host.

## Materials and Methods

### Materials

Culture media components and other reagents were of analytical grade and obtained from Sigma-Aldrich (Steinheim, Germany). Agar (a cell culture medium) was obtained from Applichem (Omaha, NE, USA). The Magnesium assay kit (BA0045) was procured from Sigma-Aldrich (Darmstadt, Germany).

### Microorganisms and Culturing Conditions

The microorganism used in this study was *Lacticaseibacillus rhamnosus* (ATCC 53103) (*L. rhamnosus)* obtained from the University of Agricultural Sciences and Veterinary Medicine in Cluj-Napoca, Romania. *L. rhamnosus* was cultured in MRS broth, which per liter contained: glucose, 20.0 g; yeast extract, 5.0 g; meat extract, 10.0 g; enzymatic digest of casein, 10.0 g; sodium acetate, 5.0 g; diammonium citrate, 2.0 g; dipotassium hydrogen phosphate, 2.0 g; manganese sulfate, 0.05 g; and Tween 80, 1.08 g, with a final pH of 6.4 ± 0.2 at 25 °C. For the control samples, the MRS medium was prepared intentionally without MgSO_4_ and autoclaved at 121 °C for 15 min [19].

For bacterial cultivation, *L. rhamnosus* was reactivated in MRS broth by transferring 1 mL of inoculum into 9 mL of sterile MRS medium in **culture flasks** (1:9 v/v). The pre-culture was incubated at 37 °C for 18–20 h to obtain actively growing cells. From this starter culture, 1 mL was subsequently inoculated into 9 mL of fresh MRS broth for experimental use. The viable cell concentration (≈ 10⁸ CFU/mL) was estimated from OD₆₀₀ measurements (Spectronic 200 spectrophotometer (Thermo Fisher Scientific, Waltham, MA, USA)) using a strain-specific calibration curve previously established for *L. rhamnosus* ATCC 53,103 correlating optical density with plate counts on MRS agar under identical culture conditions [22].

#### Fermentation, MgSO₄ optimization, and Bacterial Viability

To evaluate the effect of magnesium on *L. rhamnosus*, the bacteria were cultivated in MRS medium supplemented with four MgSO₄ concentrations (0, 0.1444, 0.722, and 1.444 g/L). These concentrations correspond to the basal Mg²⁺ level of standard MRS medium (1×) and stepwise increases up to fivefold (5×) and tenfold (10×), with 1.444 g/L representing the highest concentration. The experimental design followed a one-factor-at-a-time (OFAT) strategy, in which MgSO₄ concentration was varied while all other fermentation parameters were held constant. For inoculation, 100 µL of a culture adjusted to 10⁸ CFU/mL was added to 100 mL of MRS medium (or 400 µL into 400 mL for large-volume viability assays), and cultures were incubated at 37 °C for 18–20 h. Fermentations were carried out over 7 days, during which cultures were transferred daily into fresh medium (3500 rpm, 15 min, 21 °C) to maintain active growth. Daily samples were collected to quantify intracellular Mg²⁺ accumulation and assess bacterial viability. Viability was determined by preparing serial dilutions in sterile medium and plating 100 µL onto MRS agar [22]. Plates were incubated at 37 °C for 24–48 h, after which colony-forming units (CFU/mL) were quantified using a semi-automatic colony counter. This daily monitoring enabled evaluation of growth behavior and survival in response to different MgSO₄ concentrations. *L. rhamnosus* cultivated without supplemental MgSO₄ served as the negative control.

### Determination of Magnesium Concentration

Magnesium (Mg²⁺) content in the samples was quantified using the Magnesium Assay Kit (BA0045). The assay was performed in 96-well clear-bottom plates according to the manufacturer’s protocol. Briefly, the kit uses a calmagite dye that forms a coloured complex with Mg²⁺ and is read at 500 nm. A series of Mg²⁺ standards (0, 0.5, 1.0, 2.0 mg/dL, etc.) was prepared to generate a calibration curve for each assay. Samples (in duplicate) and standards (5 µL each) were added to the wells, followed by 200 µL of Working Reagent (Reagent A + Reagent B mixed). After 2 min of incubation at room temperature, absorbance at 500 nm (OD₁) was measured. Then 10 µL of EDTA solution was added to each well, incubated for 2 min, and absorbance was measured again at 500 nm (OD₂). The difference (OD₁ − OD₂) for each sample was interpolated onto the standard calibration curve to obtain Mg²⁺ concentration (expressed in mg/dL, then converted to µM where required). Samples exceeding the assay’s linear range (0.1–3 mg/dL) were diluted accordingly. EDTA-containing plasma samples were excluded due to chelation of Mg²⁺, as recommended by the manufacturer.

#### Assessment of Magnesium Content in Fermentations Without MgSO₄

This experiment evaluated whether *L. rhamnosus* acquires magnesium from components other than MgSO₄. For this purpose, the bacteria were grown in a modified MRS medium lacking MgSO₄. The Mg²⁺ concentration of the medium and subsequent samples was measured using the Magnesium Assay Kit (BA0045) according to the manufacturer’s instructions. For intracellular Mg²⁺ analysis, a total of 10¹⁰ cells was collected by centrifugation (8500 rpm, 2 min, 4 °C) and washed three times with phosphate-buffered saline (PBS) to remove residual extracellular magnesium. The washed biomass was resuspended in 1 mL PBS and sonicated (40% amplitude, 1 min total, 0.5-s on/off cycles), repeated 4–5 times with cooling intervals, to disrupt the cells and release intracellular magnesium. After lysis, samples were centrifuged, and the resulting supernatant was used for Mg²⁺ quantification.

#### Evaluation of Mg²⁺ Content in Bacterial Cells Cultivated with Varying MgSO_4_ Levels

This experiment assessed how different external MgSO₄ concentrations influence intracellular Mg²⁺ accumulation during a 7-day cultivation period. Cultures were grown under four MgSO₄ supplementation levels (0, 0.1444, 0.722, and 1.444 g/L). Each day, biomass equivalent to 10¹⁰ CFU was harvested and washed with phosphate-buffered saline (PBS). The washed cells were lysed by sonication under the same standardized parameters used throughout the study, and the lysates were centrifuged to separate cellular debris. The Mg²⁺ concentration in the resulting supernatant was quantified using the BA0045 assay. This procedure allowed daily monitoring of intracellular magnesium accumulation in response to different MgSO₄ supplementation levels.

### Morphological Characterization of Bacterial Cells Cultivated with Varying Concentrations of MgSO_4_

The morphological characterization of *L. rhamnosus* cells cultured with varying concentrations of MgSO_4_ was performed *via* imaging by the Electron Microscopy Core at the Max Planck Institute for Biology, Tübingen (Germany).

#### Transmission Electron Microscopy (TEM)

For transmission electron microscopy (TEM), cells were chemically fixed as before and further processed at high-pressure frozen (HPF Compact 03, Engineering Office M. Wohlwend GmbH) in capillaries. High-pressure-frozen cells were freeze-substituted (AFS2, Leica Microsystems) with 2% OsO 4 and 0.4% uranyl acetate in acetone as the substitution medium, and embedded in Epon. Ultrathin sections were stained with uranyl acetate and lead citrate and analyzed with a Tecnai Spirit (Thermo Fisher Scientific) operated at 120 kV [23].

#### Scanning Electron Microscopy (SEM)

For SEM, *L. rhamnosus* cells were fixed with 2.5% glutaraldehyde/4% formaldehyde in PBS for 3 h at room temperature, followed by overnight at 4 °C, and mounted on poly-L-lysine-coated coverslips. Then, the cells were post-fixed with 1% osmium tetroxide for 45 min on ice. Following fixation, the samples were dehydrated in a graded ethanol series and subjected to critical-point drying (CPD300, Leica Microsystems) with CO_2_. Finally, the cells were sputter-coated with a five nm-thick layer of platinum (CCU-010, Safematic) and examined with a field-emission scanning electron microscope (Regulus 8230, Hitachi High Technologies) at an accelerating voltage of 3 kV [24].

### Statistical Analysis

All experiments were performed in triplicate, and results are presented as mean ± standard deviation (SD). Statistical analyses were conducted using GraphPad Prism (Version 9.3.0, GraphPad Software Inc., San Diego, CA, USA). To assess changes in Mg²⁺ accumulation over a 7-day exposure to 1.444 g/L MgSO₄, a repeated-measures one-way ANOVA followed by Tukey’s multiple comparisons test was applied. Additionally, a two-way ANOVA with Tukey’s post hoc test was used to evaluate differences in Mg²⁺ accumulation among groups treated with varying MgSO₄ concentrations, as well as to assess bacterial viability across different MgSO₄ concentrations over time. A *p*-value of < 0.05 was considered statistically significant.

## Results

### Magnesium Uptake by *L. Rhamnosus*

LAB are pivotal in enhancing mineral bioaccessibility through multiple mechanisms. One key pathway involves the production of short-chain fatty acids (SCFAs), which lower intestinal pH and thereby increase mineral solubility, facilitating absorption [16]. Specifically, *L. rhamnosus* has been shown to modulate gut motility, potentially extending magnesium’s exposure time at the intestinal epithelium and enhancing its uptake [25]. In addition to extracellular solubilization, evidence indicates that certain *Lacticaseibacillus* species can actively internalize and accumulate magnesium within their cellular matrix via passive diffusion or specific transport systems, such as CorA-like magnesium transporters [12, 26, 27]. This intracellular sequestration may function as a transient reservoir, enabling a regulated release of magnesium into the gut microenvironment.

**Fig. 2:**
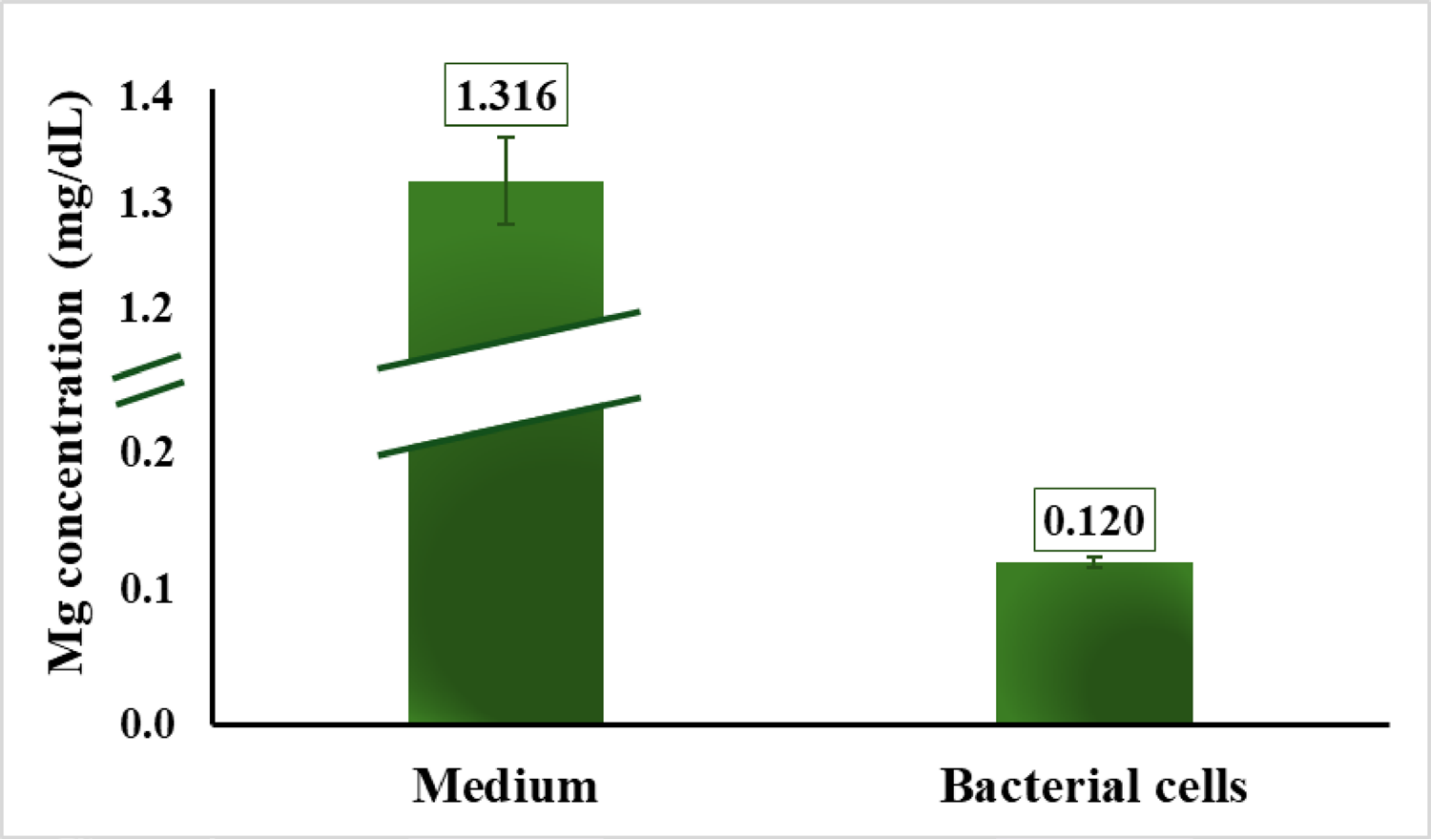
Magnesium content in MRS medium and in the supernatant obtained from lysed *L. rhamnosus* cells cultivated without MgSO₄. The supernatant reflects intracellular magnesium released after cell lysis, indicating minimal magnesium internalization by the bacteria during growth in magnesium-depleted conditions

The initial assessment focused on determining whether the MRS medium contained inherent magnesium in the absence of added MgSO₄. Analysis showed that the medium contained 1.3 mg/dL of Mg²⁺, indicating the presence of background magnesium originating from other components of the formulation (Fig. 2). Ingredients such as peptone, beef extract, and yeast extract are known to naturally contain trace minerals, including Mg²⁺ [28, 29], and are likely contributors. These observations led to a further examination of intracellular Mg²⁺ in *L. rhamnosus* cultivated under MgSO₄-free conditions (Fig. 2). After harvesting and thoroughly washing the cells to remove extracellular magnesium, the biomass was lysed, and the resulting supernatant was analyzed. Only trace Mg²⁺ levels were detected, with values approaching the assay’s lower detection limit (0.1 mg/dL).

**Fig. 3:**
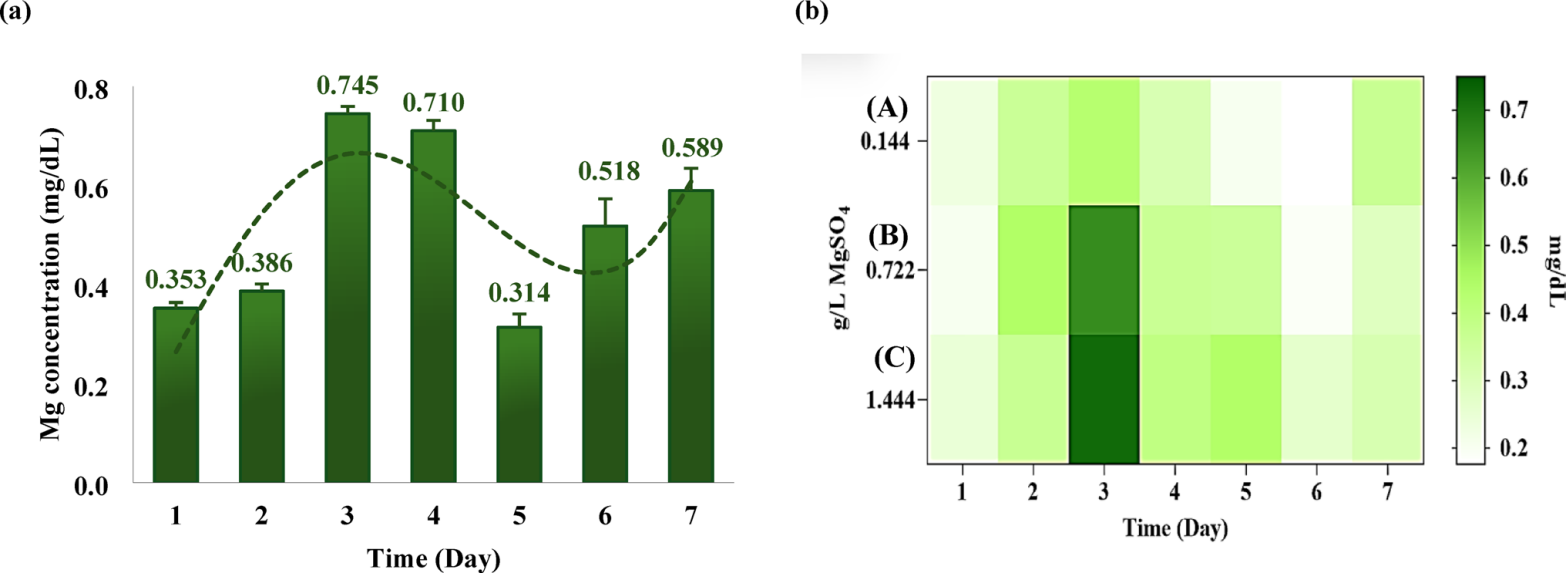
Intracellular magnesium content in *L. rhamnosus* under MgSO₄ supplementation (**a**) Left panel: Cells cultivated in medium supplemented with 1.444 g/L MgSO₄ over 7 days showed gradual intracellular magnesium accumulation with dynamic fluctuations, consistent with active uptake and homeostatic regulation. (**b**) Right panel: Intracellular Mg²⁺ levels in cells cultivated with varying MgSO₄ concentrations (0.1444, 0.722, and 1.444 g/L; Groups A–C) for 7 days. Data are expressed as mean ± SD (*n* = 3); statistical analysis (Tukey’s test) showed significant differences on day 3 between Group A vs. Group B (***) and Group A vs. Group C (***), while Group B vs. Group C was not significant (n.s.)

To examine the response of *L. rhamnosus* to elevated magnesium concentrations, the bacteria were cultivated for 7 days in a medium supplemented with a high MgSO₄ concentration (1.444 g/L, representing a 10-fold increase), with daily transfer into fresh medium containing the same supplementation (Fig. 3a). During the first two days, intracellular Mg²⁺ levels remained low. A marked increase (*p* < 0.001) occurred between days 2 and 3, with Mg²⁺ content rising from 0.3 mg/dL to 0.7 mg/dL. The concentration remained stable on day 4, subsequently decreasing to 0.3 mg/dL on day 5. Notably, Mg²⁺ levels increased again on days 6 and 7, reaching nearly 0.6 mg/dL by the end of the cultivation period.

A similar pattern of intracellular magnesium dynamics was observed when the experiment was extended to include three distinct MgSO₄ concentrations: 0.1444 g/L, 0.722 g/L, and 1.444 g/L (Fig. 3b). *L. rhamnosus* did not exhibit significant magnesium accumulation in any samples during the initial two days. The highest values recorded during this period were 0.4 mg/dL at 0.722 g/L (Group B) and 0.3 mg/dL at 1.444 g/L (Group C). By day 3, however, intracellular magnesium content increased markedly, reaching 0.7 mg/dL in cultures exposed to 1.444 g/L MgSO₄ and 0.65 mg/dL in those grown at 0.722 g/L. Throughout the remaining days of the experiment, Mg²⁺ accumulation fluctuated across all tested concentrations.

### Growth Kinetics of *L. Rhamnosus* Under Magnesium Supplementation

**Fig. 4:**
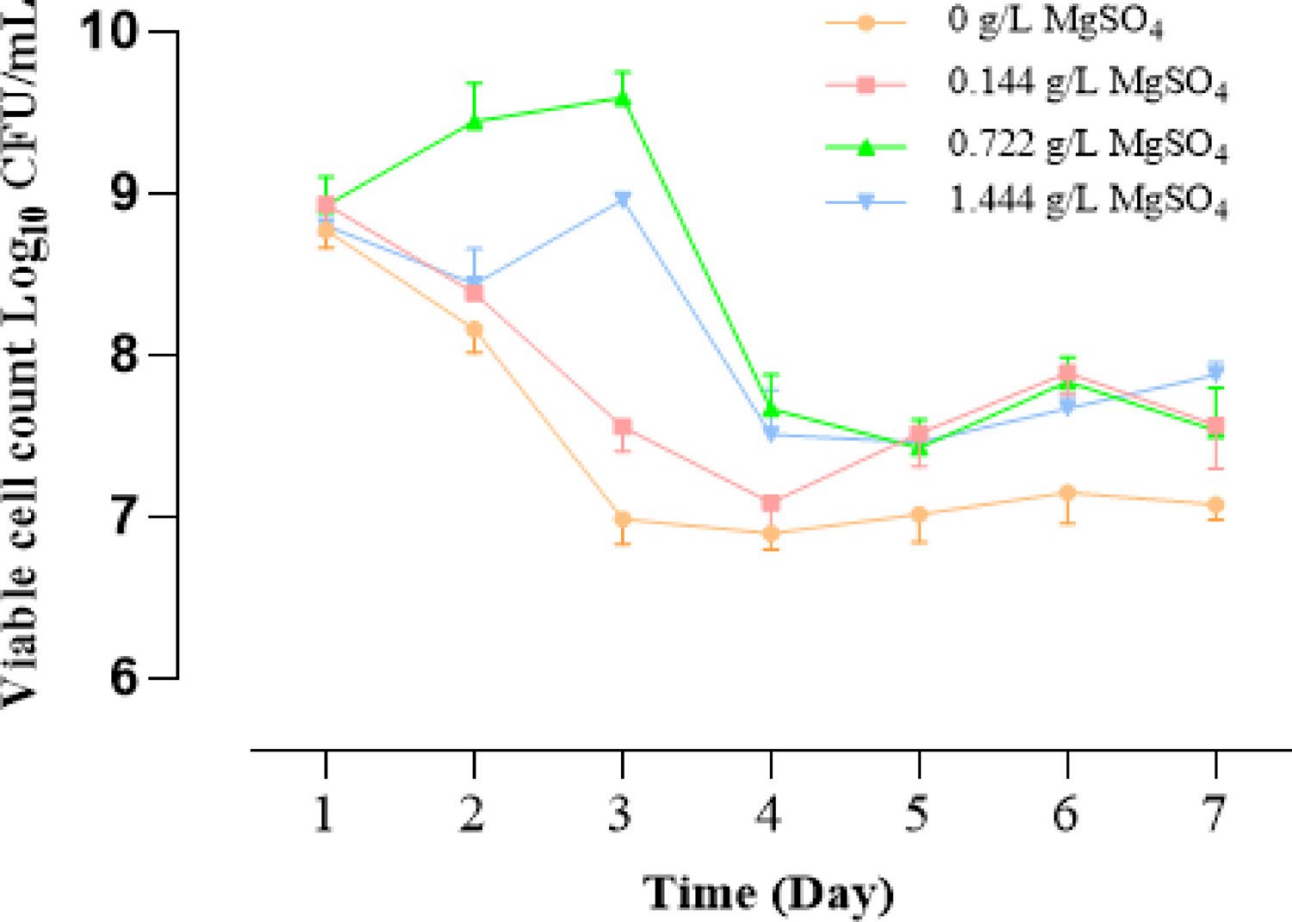
Viability of *L. rhamnosus* over 7 days under different MgSO₄ concentrations. Values are expressed as log₁₀ CFU/mL (mean ± SD, *n* = 3). Statistical analysis was performed in GraphPad Prism 9.3.0. using two-way ANOVA followed by Tukey’s multiple comparisons test. Significant differences were found between all groups (***p < 0.001), except for 0.722 vs. 1.444 g/L (***p = 0.001) (Supplementary file)

Magnesium (Mg²⁺) is an essential divalent cation involved in numerous cellular processes, including DNA replication, RNA transcription, ribosome stability, enzyme activity, and membrane integrity. In LAB, including *L. rhamnosus*, Mg²⁺ is essential for supporting active transport systems, ATP synthesis, and stress tolerance [30]. The results following the investigation of the effect of different concentrations of MgSO₄ on the growth of *L. rhamnosus* revealed an apparent dose-dependent influence of Mg²⁺ on CFU/mL values, with the greatest growth enhancement observed at 0.722 g/L MgSO₄. This concentration led to an exponential increase in viable cell count between Day 1 and Day 3, peaking at 2.6 × 10⁹ CFU/mL, suggesting this is an optimal concentration for maximizing biomass yield under the given conditions (Fig. 4). Additionally, the absence of Mg²⁺ led to a sharp, sustained decrease in CFU/mL after Day 1, likely due to depletion of internal Mg²⁺ pools and the inability to support DNA replication, membrane repair, and other vital processes [31]. The low concentration of MgSO₄ (0.1444 g/L) allowed modest recovery after Day 3, while the highest concentration tested (1.444 g/L) resulted in moderate growth but better long-term survival, as evidenced by the highest CFU/mL on Day 7. This suggests that elevated Mg²⁺ levels may stabilize membranes or mitigate acid stress, consistent with divalent cations’ buffering capacity and osmoprotective roles [32].

### Morphological Characterization of Bacterial Cells Cultivated with Varying Concentrations of MgSO_4_

**Fig. 5:**
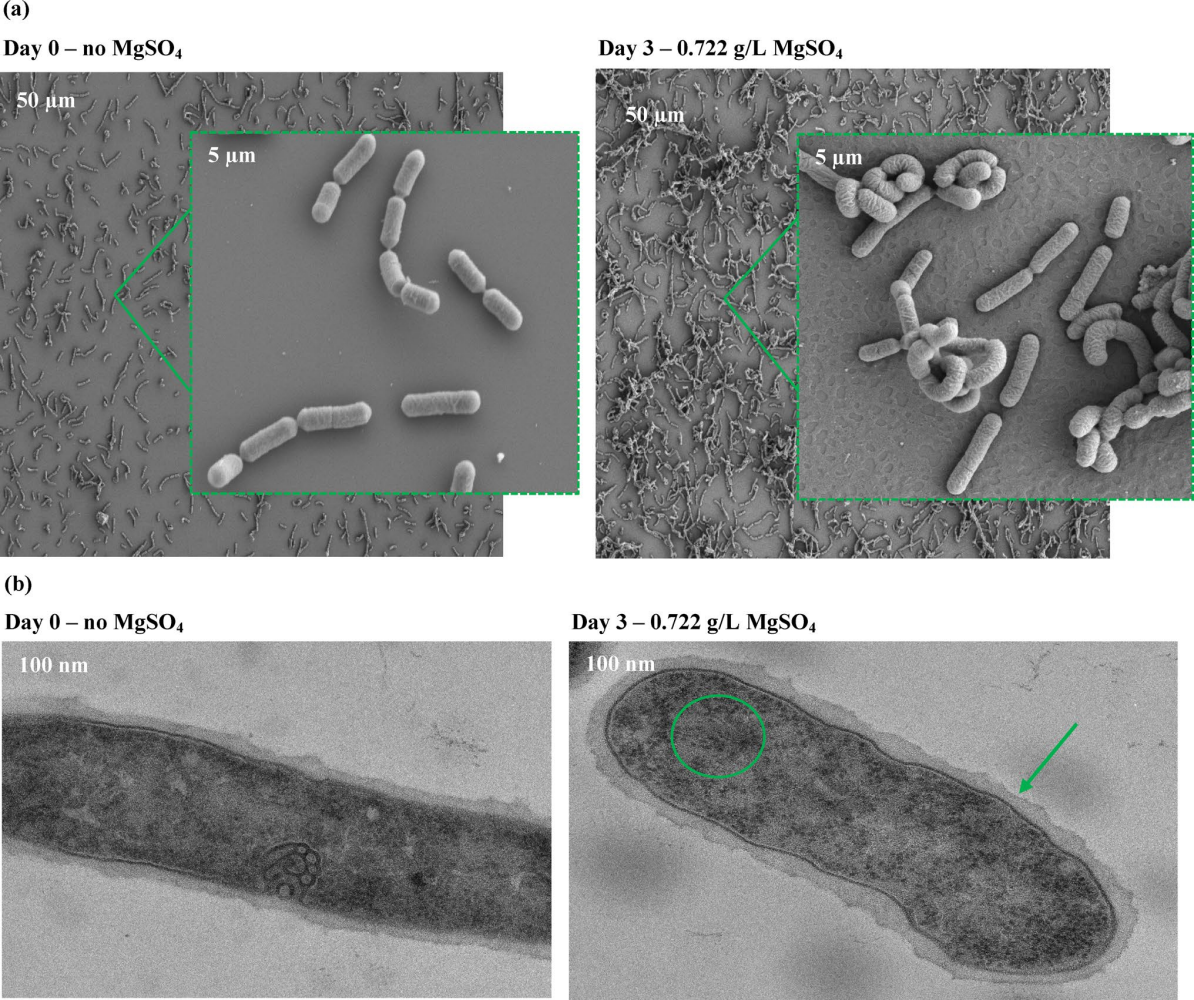
Representative SEM **(a)** and TEM **(b)** images of *Lacticaseibacillus rhamnosus* ATCC 53,103 cells cultivated without MgSO_4_ and with 0,722 g/L MgSO_4_. **(a)** SEM micrographs obtained with a Hitachi Regulus 8230 field-emission scanning electron microscope operated at 3 kV (resolution ≈ 1–2 nm). Scale bars = 50 μm and 5 μm; corresponding magnifications ≈ ×2,500 and ×25,000, respectively. **(b)** TEM image acquired on a Tecnai G2 Spirit BioTwin transmission electron microscope (Thermo Fisher Scientific) operated at 120 kV, with a resolution ≈ of 0.34 nm. Scale bar = 100 nm (magnification ≈ ×120,000)

In this section, we characterize *L. rhamnosus* cells grown with 0.722 g/L MgSO₄ and without any addition of Mg²⁺ salts using Scanning Electron Microscopy (SEM) and Transmission Electron Microscopy (TEM). Our primary goal was to explore the role of *L. rhamnosus* as a potential internalizer of Mg²⁺. The SEM images provide insights into surface morphology and cell aggregation, while TEM images reveal detailed internal cellular structures, such as cell wall integrity and intracellular organization (Fig. 5). By comparing the structural differences between cells grown in the presence and absence of MgSO₄, we aimed to shed light on how MgSO₄ influences the physical and structural properties of *L. rhamnosus* and assess its capacity for Mg²⁺ uptake and internalization.

## Discussion

Our results show that *L. rhamnosus* internalizes magnesium in a concentration- and time-dependent manner, but in a strictly regulated way. In the absence of MgSO₄ supplementation, residual magnesium from medium components such as peptone or yeast extract was not bioaccessible, and intracellular Mg²⁺ remained near the detection threshold. This finding suggests that uptake depends on chemical availability and also on biological control of transport activity. CorA-family transporters are known to be the primary Mg²⁺ uptake systems in LAB, and their expression is tightly controlled by extracellular magnesium levels [31, 33]. The lag phase observed during the first two days of cultivation, followed by a sharp increase on day 3, strongly suggests that *L. rhamnosus* upregulates high-affinity transport only when persistent external Mg²⁺ availability is detected. Such regulatory gating prevents unnecessary transporter expression under transient conditions and protects cells from ionic overload [31, 34].

The subsequent fluctuations in intracellular Mg²⁺, with a peak followed by partial depletion and later recovery, indicate a homeostatic cycle of uptake, utilization, and efflux. Magnesium is indispensable for stabilizing nucleic acids, activating enzymes, and fueling ATP metabolism. ATP and rRNA represent the largest intracellular Mg²⁺ reservoirs, and redistribution into these pools could account for the observed decreases after day 3 [31, 33]. Efflux may also contribute, as many bacteria possess Mg²⁺ exporters that prevent excess accumulation and maintain osmotic balance. The delayed but enhanced uptake at 0.722 and 1.444 g/L MgSO₄ supports the view that transporter induction is concentration dependent and that uptake capacity increases only after a regulatory threshold is reached. Together, these dynamics emphasize that magnesium handling in *L. rhamnosus* is an actively regulated process rather than a passive consequence of external availability [34].

Growth kinetics assays reinforced the centrality of magnesium for probiotic physiology. In magnesium-free conditions, viability dropped sharply after one day, reflecting the exhaustion of internal Mg²⁺ reserves needed for replication, transcription, and protein synthesis [31]. Supplementation prevented this effect, but in a dose-dependent manner. At 0.722 g/L MgSO₄, exponential growth was maximized, reaching 2.6 × 10⁹ CFU/mL by day 3, indicating this concentration optimally supports DNA replication, ribosome assembly, and enzymatic activity. At 1.444 g/L, growth was initially slower, but long-term survival was better than at lower concentrations, consistent with divalent cations enhancing membrane stability and acid stress tolerance [32]. These findings are consistent with earlier studies in LAB, where magnesium limitation impaired acid resistance and supplementation improved viability [35]. From a probiotic perspective, the ability of *L. rhamnosus* to thrive under magnesium-enriched conditions suggests it can serve as both a functional probiotic and a carrier of bioaccessible magnesium [12].

Morphological analyses provide further evidence of dual localization of magnesium in *L. rhamnosus*. SEM revealed surface modifications in cells grown with 0.722 g/L MgSO₄, with ruffled cell walls compared to the smooth surfaces of Mg-deprived cells. These changes can be attributed to the electrostatic binding of Mg²⁺ to negatively charged peptidoglycan and teichoic acids [30, 32]. At low occupancy, this binding stabilizes the cell wall, but as sites become saturated, affinity decreases, and additional ions are held more loosely [32]. This mechanism implies that magnesium-loaded bacteria could later release Mg²⁺ back into the environment, particularly in the intestine, where competing ions and lower ambient Mg²⁺ levels favor desorption. TEM further revealed more prominent ribosomes in Mg-supplemented cells, consistent with intracellular magnesium sequestration in rRNA and ATP-binding pools [12, 31]. Together, these ultrastructural findings suggest a two-compartment storage strategy: tightly bound intracellular magnesium supporting metabolism and loosely bound cell wall-associated magnesium that may act as a releasable reservoir. In our experiment, 0.7 mg Mg²⁺/dL was detected in the supernatant obtained after lysis of 10⁹ *L. rhamnosus cells*, corresponding to 0.007 mg Mg²⁺/mL. Considering that 10⁹ CFU typically corresponds to ~ 1 mg dry biomass, this equates to an intracellular content of approximately 7 mg Mg²⁺ per gram of dry biomass [36]. This value indicates that enrichment under conventional cultivation conditions was in fact higher than the levels reported in PEF-assisted experiments, where *L. rhamnosus* B 442 accumulated up to 4.28 mg/g, and other LAB strains reached ~ 1.8–2.0 mg/g [12, 35]. The difference may reflect strain-specific responses or methodological factors, but it also suggests that conventional supplementation can yield substantial magnesium accumulation in *L. rhamnosus*.

Taken together, these findings support the concept of *L. rhamnosus* as a dual-function probiotic: it contributes to gut health through established probiotic mechanisms and simultaneously acts as a bio-carrier for magnesium. Surface-bound Mg²⁺ may be gradually released in the gut, while intracellular magnesium supports bacterial metabolism and potentially enriches the local microenvironment. Such microbe-mediated mineral delivery could complement traditional supplementation, particularly in populations with magnesium deficiency. Although our findings strongly suggest that magnesium uptake in *L. rhamnosus* involves CorA-family transporters, this conclusion is based on indirect evidence derived from physiological and morphological responses. We did not perform direct molecular analyses (e.g., quantification of corA gene expression or transporter protein levels) to confirm their involvement. Therefore, this interpretation should be viewed as a mechanistic hypothesis supported by observed uptake patterns rather than definitive proof of CorA activation. Future studies integrating transcriptomic and proteomic profiling under different Mg²⁺ conditions could validate the regulatory role of CorA and related efflux systems. Such work would also clarify whether the observed magnesium fluctuations reflect transcriptional control, post-translational modulation of transporter activity, or contributions from additional Mg²⁺ transport pathways [37, 38].

## Conclusions

Magnesium is an essential micronutrient required for numerous physiological processes, yet its bioavailability from dietary sources often remains suboptimal. In this study, *Lacticaseibacillus rhamnosus* demonstrated the capacity to internalize magnesium under varying MgSO₄ concentrations, with intracellular levels reaching ~ 7 mg/g dry biomass.SEM and TEM analyses revealed that magnesium associates both with the cell wall, likely via electrostatic binding to peptidoglycan and teichoic acids, and intracellularly, predominantly within ribosomal and nucleic acid structures. These findings indicate a dual compartmentalization strategy that may enable *L. rhamnosus* to act as a magnesium reservoir, with the potential to release bioaccessible Mg²⁺ in the intestinal environment.

Additionally, these results highlight the feasibility of magnesium enrichment in probiotics; the functional consequences for host absorption and health remain to be clarified. The discrepancy with PEF-based studies suggests that strain-specific traits, extended cultivation with repeated Mg²⁺ exposure, and differences in biomass normalization may influence accumulation efficiency. Future studies should integrate transcriptomic and proteomic analyses of magnesium transporters, as well as in vitro assays using Caco-2 cells and in vivo models, to confirm the bioaccessibility and physiological impact of probiotic-mediated magnesium delivery. Nonetheless, this research lays the groundwork for future nutribiotic strategies to improve mineral uptake via probiotic carriers.

## Supplementary Information

Below is the link to the electronic supplementary material.

Supplementary Material 1

## Data Availability

No datasets were generated or analysed during the current study.

## Declarations

### **Generative AI and AI-assisted Technologies in the Writing Process**

During the preparation of this work, the authors used Open AI and ChatGPT − 4 to improve its readability and language.
